# Lung and Pleural Immunoglobulin G4-Related Disease: Two Contrasting Case Reports

**DOI:** 10.7759/cureus.35439

**Published:** 2023-02-25

**Authors:** Mariana Diz-Lopes, Fernando Nogueira, José Alberto da Cunha Marques, Sofia Pedrosa, Carlos Marques-Gomes, Teresa Martins-Rocha, Pedro Von-Hafe, Conceição Souto-Moura

**Affiliations:** 1 Rheumatology, Centro Hospitalar Universitário de São João, Porto, PRT; 2 Internal Medicine, Centro Hospitalar Universitário de São João, Porto, PRT; 3 Pathology and Laboratory Medicine, Centro Hospitalar Universitário de São João, Porto, PRT

**Keywords:** aortitis, pleural diseases, lung diseases, immunoglobulin g4-related disease, immunoglobulin g

## Abstract

Immunoglobulin G4-related disease (IgG4-RD) is an immunomediated disease that can virtually affect any organ. Despite the pancreas being known as the most frequently involved organ, pulmonary and pleural IgG4-RD is being increasingly reported. The authors present two cases of IgG4-RD diagnosed in the same year, with different presentations and outcomes, in which the lung and pleural involvement were essential for the diagnosis. Recognizing IgG4-RD as a possible cause of chronic pleural effusion and/or thickening and lung abnormalities is important for an early diagnosis and prognosis improvement.

## Introduction

Immunoglobulin G4-related disease (IgG4-RD) is a recently recognized condition, with the first report dating two decades ago in Japan in a group of patients with autoimmune pancreatitis in which elevated serum Immunoglobulin G4 (IgG4) levels were identified [1]. A few years later, it was acknowledged as a systemic condition, when several extra-pancreatic manifestations were identified in these patients [2]. IgG4-RD is a chronic fibroinflammatory systemic disease, and its epidemiology and etiology are still uncertain. However, there is a clear male predominance, with a mean age at diagnosis of approximately 60 years. Involvement of major organs is frequent, with the pancreas, liver, and biliary tree being the mostly reported as well as major salivary and lacrimal glands [3]. IgG4-related lung and pleural disease is less common, but it is being increasingly reported, either in isolation or in association with other organ involvement. Radiologic lung manifestations can include nodules, ground-glass opacities, interstitial disease, and/or bronchovascular bundle thickening. In the pleura, pleural diffuse or nodular thickening is common, but pleural effusions are less frequently observed [3-5]. Diagnostic workup of IgG4-related lung and pleural disease can be challenging as it can easily mimic other etiologies such as malignancy or granulomatous diseases, which can also present with elevated IgG4 serum levels [4,6,7]. Histopathological findings can therefore be the key to diagnosis. The pathological hallmarks of the disease, which are similar independent of the location, include lymphoplasmacytic infiltration, obliterative phlebitis, storiform fibrosis, and IgG4-positive plasma cell infiltration. The diagnosis of IgG4-RD depends on the coexistence of clinical, serological, radiological, and histopathological evidence, and none will be sufficient by itself [8]. Glucocorticoids are the first-line treatment for the induction of remission of IgG4-RD. Diagnosis and early treatment of these patients are essential as there is usually a good response to treatment, and it can prevent progression to irreversible fibrosis and organ dysfunction [7,9,10].

## Case presentation

Case 1

A 78-year-old man presented to the emergency department with a 15-day history of worsening dyspnea, generalized weakness, and anorexia. He had cardiovascular and respiratory symptoms reported for over 10 years, with several admissions to the hospital. One of them was because of constrictive pericarditis, in which he was submitted to a partial pericardiectomy, with a histopathological exam that only revealed lymphoplasmacytic infiltrate, but no immunostaining for IgG4 plasma cells was performed at that time. The other admissions were related to chronic pleural effusion and pleural thickening, and the patient was submitted to multiple thoracocentesis and one pleural biopsy. The bacterial and mycological culturing were negative as well as polymerase chain reaction (PCR) and culturing for mycobacteria. Malignancy was excluded, and histopathological examination showed a polymorphic inflammatory infiltrate. During this period, he had numerous infectious intercurrences, having taken several antibiotics.

**Figure 1:**
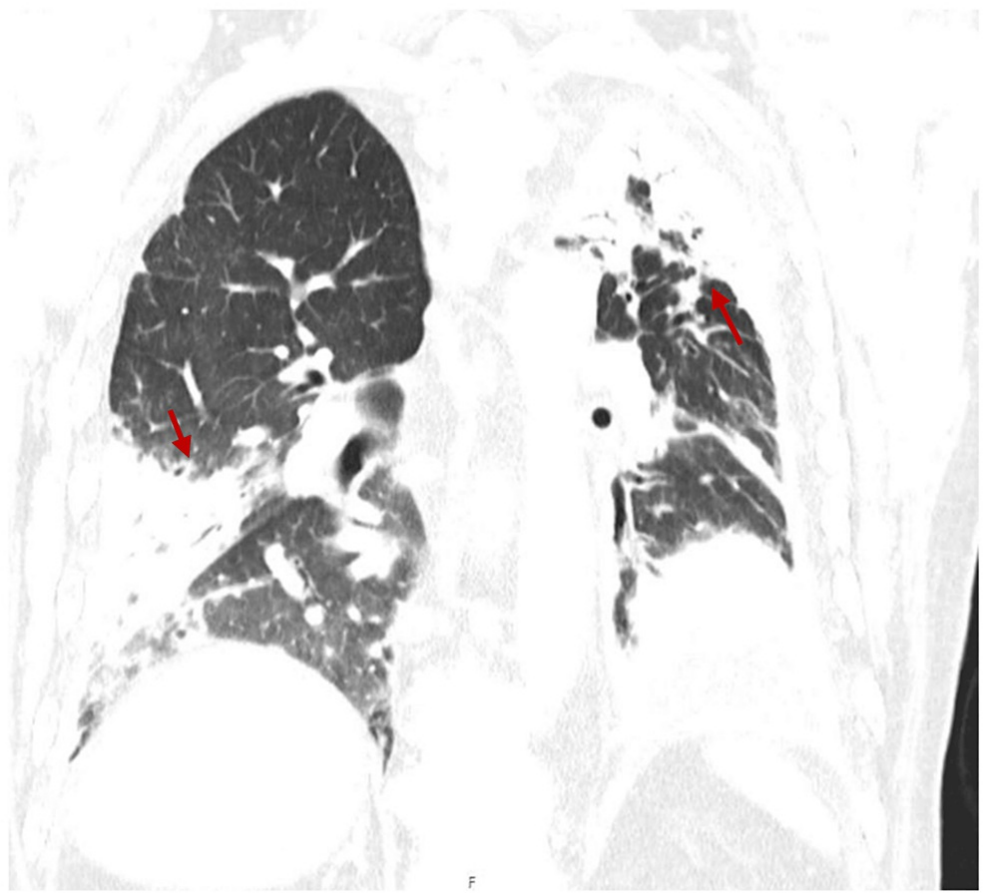
Chest computed tomography (CT) scan of Patient 1 on admission showing bilateral pleural effusion with pleural thickening and extensive areas of ground-glass appearance on pulmonary parenchyma, mainly on the left side (red arrows)

On admission, the physical examination was remarkable for hypoxemia, decreased breath sounds at the left lung base, and symmetric peripheral edema. Electrocardiogram showed atrial multifocal rhythm. Laboratory blood tests showed C-reactive protein (CRP) of 22.7 mg/L, erythrocyte sedimentation rate (ESR) of 24 mm/h, positive antinuclear antibodies (ANAs) titers (1/1000) in a speckled pattern, consumption of serum complement proteins (C3c and C4), marked elevation of serum levels of total IgG (3210 mg/dL, reference range: 600-1560 mg/dL), IgG4 (2100 mg/dL, reference range: 8-140 mg/dL), and IgE (331 kU/L, reference range: <114 kU/L). Other autoantibodies tests were normal. A computed tomography (CT) scan of the thorax was performed, revealing stability of the chronic pleural effusion and pleural thickening on the left, with newly identified pleural effusion and pleural thickening on the right as well as the progression of extensive areas of ground-glass appearance on pulmonary parenchyma (Figure 1).

**Figure 2:**
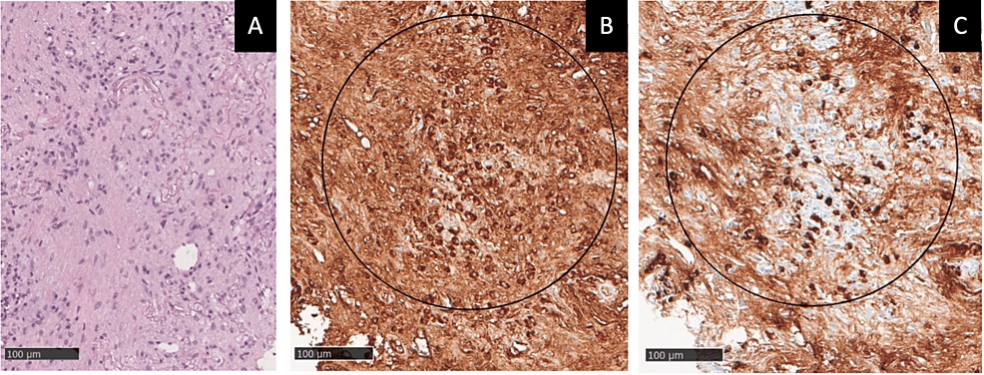
Histopathological findings of lungs in Patient 1 (A) Hematoxylin-eosin (HE) stain, 100x: stromal fibrosis with moderate lymphoplasmacytic cell infiltration of the interstitium. (B) Immunostaining, 400x: immunoglobulin (IgG)-positive plasma cells. (C) Numerous immunoglobulin G4 (IgG4)-positive plasma cells: >20 IgG4-positive cells/high power field (HPF) and IgG4/IgG-positive cell ratio of 40%-45%.

Also, there were several new lymphadenopathies (hilar, mediastinal, pericardiophrenic, and supraclavicular). An 18F-fluorodeoxyglucose-positron emission tomography (18F-FDG PET) showed increased uptake in some areas of the pulmonary parenchyma and in the pleura, with no uptake in other organs. After a transthoracic pulmonary biopsy, the histopathologic study revealed the presence of a moderate lymphoplasmacytic infiltrate and more than 20 IgG4 positive plasma cells per high power field (HPF) with an IgG4/IgG-positive cell ratio of 40%-45% (Table 1, Figure 2).

**Table 1:** Diagnostic criteria for IgG4-related respiratory disease: Two or more of the histological items are required from intrathoracic organ tissues Commentary: For the pathological diagnosis, the use of surgical biopsy specimens is desirable. In our report, the specimens evaluated were transthoracic biopsies. Such small samples make the evaluation of the fibrotic pattern very difficult [11].

Histological criteria	Case 1	Case 2
(1) Marked lymphoplasmacytic cell infiltration into the interstitium of peribronchovascular sheath, interlobular septal wall, and/or pleura	Moderate lymphoplasmacytic cell infiltration of the interstitium	Dense lymphoplasmacytic cell infiltration of the interstitium with occasional eosinophils
(2) IgG4/IgG-positive cell ratio > 40% and/or > 10 IgG4-positive cells/high power field (HPF)	IgG4/IgG-positive cell ratio: 40%-45% and IgG4-positive cells/HPF: >20	IgG4/IgG-positive cell ratio: 70%-75% and IgG4-positive cells/HPF: >50
(3) Obliterative phlebitis or obliterative arteritis	Not identified	Not identified
(4) Storiform fibrosis or fibrosis consisting of proliferating spindle-shaped cells around infiltrating lymphocytes	Stromal fibrosis with occasional spindle cell proliferation (without atypia)	Hyaline fibrosis replacing the normal parenchyma

**Figure 3:**
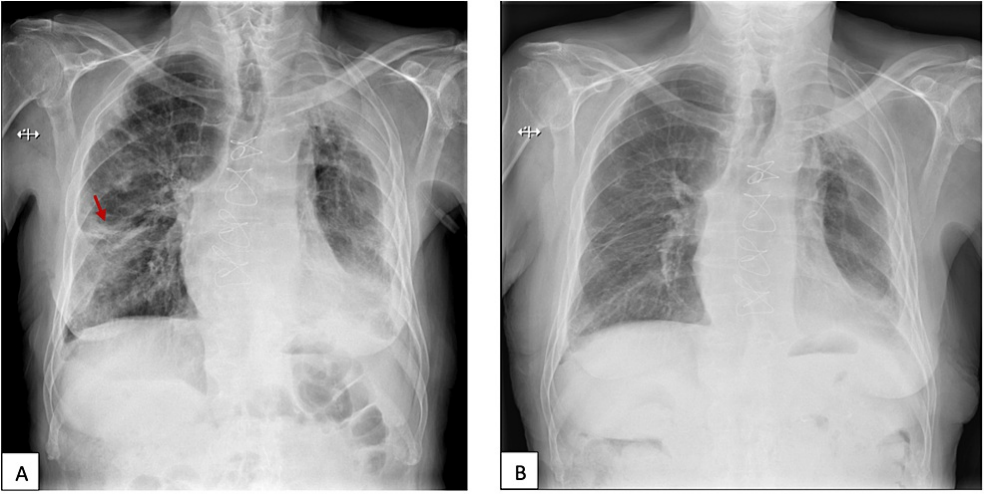
Chest radiographs of Patient 1 on admission (A) and one month after corticosteroids (B) It shows regression of the lung infiltrate on the right after a month of treatment with corticosteroids (red arrow) and stable pleural effusion on the left.

Given the diagnostic possibility of IgG4-RD, a histopathological reevaluation of the pericardial biopsy was requested, but it only revealed 15 IgG4-positive plasma cells per HPF but with an IgG4/IgG plasma cell ratio of less than 40%. The diagnosis of IgG4-RD was made, and the patient was started on 40 mg (0.5 mg/kg) of prednisolone. A month later, he was evaluated in an outpatient setting: Chest radiography showed regression of the lung nodules and stable pleural effusion (Figure 3), and blood tests revealed decreasing levels of IgG4 (from 2100 to 580 mg/dL) as well as normal CRP and ESR. However, he still reported dyspnea and anorexia. He died a couple of weeks later of an unknown cause.

Case 2

**Figure 4:**
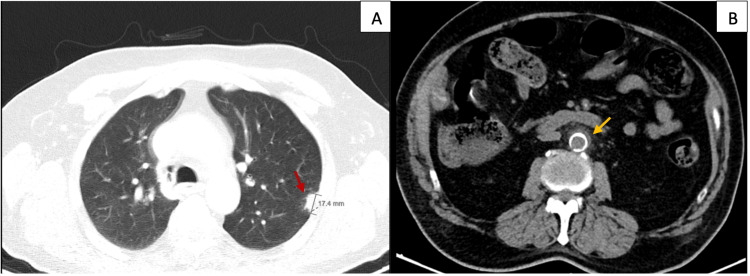
Computed tomography (CT) scan of Patient 2. (A) Chest CT scan revealing one of the newly identified pleural nodules on the left inferior lobe (red arrow). (B) Abdominal CT scan with periaortitis (densification of the fat adjacent to the infrarenal aorta, yellow arrow).

A 78-year-old man was admitted to the hospital with a 10-day history of fever and inflammatory signs on the left foot. His past medical history was significant for type 2 diabetes mellitus, chronic arterial hypertension, and chronic renal disease on a current hemodialysis program. He was started on antibiotics for cellulitis, with good clinical improvement and fever resolution but sustained elevation of CRP (400 to 200 mg/L) and ESR (128 mm/h). Other laboratory blood tests were requested, showing normocytic normochromic anemia, positive ANA titers (1/100) in a speckled pattern with negativity for other autoantibodies, normal serum levels of complement proteins, normal serum levels of total IgG, and elevation of IgG4 serum levels (IgG4: 201 mg/dL, reference range: 8-140 mg/dL). A whole-body CT scan showed two new pleural nodules and densification of the fat adjacent to the infrarenal aorta with a longitudinal extension of 47 mm on a circumferential pattern (Figure 4).

**Figure 5:**
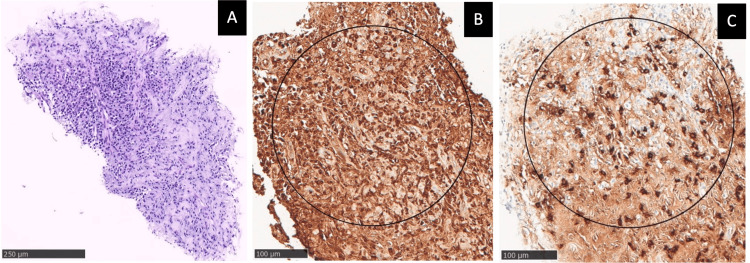
Histopathological findings of the pleura of Patient 2 (A) Hematoxylin-eosin (HE) stain, 100x: hyaline fibrosis replacing the normal parenchyma. (B) Immunostaining, 400x: immunoglobulin (IgG)-positive plasma cells. (C) Numerous immunoglobulin G4 (IgG4)-positive plasma cells: >50 IgG4-positive cells/high power field (HPF) and IgG4/IgG-positive cell ratio of 70%-75%.

An 18-FDG PET had increased uptake in the abdominal infrarenal aorta and in the pleural nodular thickening. A transthoracic pleural biopsy was performed, and the histopathologic study revealed the presence of a dense lymphoplasmacytic infiltrate and more than 50 IgG4-positive plasma cells per HPF with an IgG4/IgG-positive cell ratio of 70%-75% (Table 1, Figure 5). The aspects were compatible with a diagnosis of IgG4-RD, and even with the acknowledgment of the current absence of symptoms, he was started on 40 mg (0.5 mg/kg) of prednisolone. After four weeks of treatment, he was still asymptomatic with no new complaints. No reevaluation with imaging studies had been performed yet, but CRP had significantly decreased.

## Discussion

IgG4-RD can present with lung nodules, ground-glass opacities, bronchovascular bundle thickening, pleural effusions, or pleural thickening. Even though lung and pleural involvement is an infrequent manifestation (12% cases) of IgG4-RD, encountering patients with lung nodules and pleural effusions is common in clinical practice [3].

The first case reports a 10-year evolution of unilateral pleural effusion and pleural thickening with later progression to both sides, associated with pulmonary nodules that were labeled as idiopathic for several years, underlying the challenge of the diagnosis of IgG4-RD. Although the diagnosis results from the integration of clinical, radiological, serological, and histopathological features, histology is rarely the key to the diagnosis. Comprehensive diagnostic criteria have been published in 2020 with the goal of helping clinicians make an accurate diagnosis, but tissue samples are usually necessary to exclude malignancy [12]. In this case, previous pericardial and pleural biopsies were insufficient for the diagnosis, and immunostaining for IgG4 plasma cells had not been performed. The transthoracic pulmonary biopsy was essential as it showed the typical lymphoplasmacytic infiltrate and positive immunostaining for IgG4 plasma cells. This patient had IgG4-RD with definite pulmonary involvement and probable pericardial and pleural involvement.

In the second case, the diagnosis was possible after a transthoracic pleural biopsy. Aortitis and periaortitis are also reported manifestations of IgG4-RD and are associated with significant morbidity. Radiologically, they present as an arterial wall or periarterial tissue thickening in a circumferential or anterolateral pattern. Abdominal aorta periaortitis is the most commonly encountered manifestation. However, obtaining tissue samples, given the location, can be challenging [13].

IgG4-RD usually responds well to treatment with glucocorticoids. Treatment is recommended in most patients, even when asymptomatic, as it is documented to induce remission and slow progression to fibrosis and organ dysfunction. A delay in treatment can cause irreversible sequelae [14]. A good response to treatment is achieved if there is an improvement in the overall clinical status, a decrease in serum IgG4 concentration, and an improvement in the previously identified radiologic abnormalities. To help evaluate treatment response, an IgG4-RD responder index has been developed [14,15]. Most patients with IgG4-RD with lung and pleural involvement have no symptoms as in the second case [4,6]. The first patient had dyspnea, anorexia, and generalized weakness related to not only lung and pleural involvement of IgG4-RD but also cardiac dysfunction. He showed partial response to therapy in the first consultation after discharge as he remained significantly symptomatic but had analytical and radiological improvement. It has been described that in some patients, long-standing fibrotic lesions respond poorly, if at all, to glucocorticoids. Steroid-sparing agents (azathioprine, mycophenolate mofetil, methotrexate, tacrolimus, cyclophosphamide, and 6-mercaptopurine) may be required during treatment due to the inefficacy of glucocorticoids or side effects associated with its chronic use. Rituximab is also an effective treatment option [14]. This patient's death shortly after starting glucocorticoids highlights the importance of early diagnosis and treatment.

## Conclusions

Raising awareness for IgG4-related lung and pleural disease is of major importance. It is an important differential diagnosis in patients with lung parenchyma abnormalities, pleural effusion, and/or thickening, particularly when there are elevated serum IgG4 levels. It can occur in isolation or in conjunction with other organ manifestations, but transthoracic pulmonary or pleural biopsy is the safest and easiest way to obtain tissue samples, and it is frequently available, enabling the exclusion of other diseases and the definite diagnosis. Given the risk of progressing to permanent fibrosis, it is crucial to provide timely treatment for these patients to improve overall survival.

## References

[1] Hamano H, Kawa S, Horiuchi A (2001). High serum IgG4 concentrations in patients with sclerosing pancreatitis. N Engl J Med.

[2] Kamisawa T, Funata N, Hayashi Y (2003). A new clinicopathological entity of IgG4-related autoimmune disease. J Gastroenterol.

[3] Brito-Zerón P, Ramos-Casals M, Bosch X, Stone JH (2014). The clinical spectrum of IgG4-related disease. Autoimmun Rev.

[4] Ryu JH, Sekiguchi H, Yi ES (2012). Pulmonary manifestations of immunoglobulin G4-related sclerosing disease. Eur Respir J.

[5] Fei Y, Shi J, Lin W (2015). Intrathoracic involvements of immunoglobulin G4-related sclerosing disease. Medicine (Baltimore).

[6] Naramala S, Biswas S, Adapa S, Gayam V, Castillo RC, Annangi S, Konala VM (2019). Pleomorphic pulmonary manifestations of IgG4-related disease. Case Rep Rheumatol.

[7] Morales AT, Cignarella AG, Jabeen IS, Barkin JS, Mirsaeidi M (2019). An update on IgG4-related lung disease. Eur J Intern Med.

[8] Wallace ZS, Naden RP, Chari S (2020). The 2019 American College of Rheumatology/European League Against Rheumatism Classification Criteria for IgG4-related disease. Arthritis Rheumatol.

[9] Brito-Zerón P, Bosch X, Ramos-Casals M, Stone JH (2016). IgG4-related disease: advances in the diagnosis and treatment. Best Pract Res Clin Rheumatol.

[10] Ardila-Suarez O, Abril A, Gómez-Puerta JA (2017). IgG4-related disease: a concise review of the current literature. Reumatol Clin.

[11] Matsui S, Yamamoto H, Minamoto S, Waseda Y, Mishima M, Kubo K (2016). Proposed diagnostic criteria for IgG4-related respiratory disease. Respir Investig.

[12] Umehara H, Okazaki K, Kawa S (2021). The 2020 revised comprehensive diagnostic (RCD) criteria for IgG4-RD. Mod Rheumatol.

[13] Nikiphorou E, Galloway J, Fragoulis GE (2020). Overview of IgG4-related aortitis and periaortitis. A decade since their first description. Autoimmun Rev.

[14] Khosroshahi A, Wallace ZS, Crowe JL (2015). International Consensus Guidance statement on the management and treatment of IgG4-related disease. Arthritis Rheumatol.

[15] Carruthers MN, Stone JH, Deshpande V, Khosroshahi A (2012). Development of an IgG4-RD responder index. Int J Rheumatol.

